# The vasodilatory effect of *Apium graveolens *L (celery) seed in isolated rat aorta: The roles of endothelium, calcium and potassium channels 

**Published:** 2021

**Authors:** Farzaneh Sohrabi, Saeed Niazmand, Maryam Mahmoudabady, Mohammad Javad Niazmand

**Affiliations:** 1 *Department of Physiology, Faculty of Medicine, Mashhad University of Medical Sciences, Mashhad, Iran*; 2 *Neurogenic Inflammation Research Center, * *Mashhad University of Medical Sciences, Mashhad, Iran*; 3 *Department of Health Sciences, McMaster University, Hamilton, Canada*

**Keywords:** Apium graveolens, Isolated aorta, Vasorelaxation, Calcium channels, Potassium channels

## Abstract

**Objective:**

*Apium graveolens L.* (celery) seed has been used for hypertension treatment. To provide a pharmacological basis, the vasorelaxant effect of celery seed extract was investigated in isolated rat aorta.

**Materials and Methods:**

Wistar male rats (200-250 g) were divided into 15 groups (n=7 for each group). The vasorelaxant response of different concentrations of celery seed extract (0.05, 0.1, 0.25, 0.5, 1, and 2 mg/ml) on isolated aorta precontracted with phenylephrine (PE) or KCl was evaluated by organ bath technique. The role of endothelium, extracellular calcium influx, intracellular sources of calcium, and potassium channels in vasorelaxant effect of celery seed extract was investigated.

**Results:**

The extract showed a concentration-dependent relaxation in the isolated aorta contracted with PE and KCl that was endothelium-dependent at lower concentrations. Pretreatment of aortic rings with indomethacin or L-NAME, did not affect the vasorelaxation induced by celery seed extract. The extract inhibited KCl and PE-induced contractions in cumulative calcium concentrations as well as after incubation with diltiazem in denuded aortic rings of endothelium. The relaxation induced by celery seed extract was inhibited by 4-aminopyridine.

**Conclusion:**

This relaxation was mediated by inhibiting calcium influx into vascular smooth muscle cells. Also, voltage-dependent potassium channels were involved in inducing the vasorelaxant effect of celery seed extract.

## Introduction

*Apium graveolens L*, commonly called celery, is a popular and common vegetable throughout the world (Yang et al., 2019). Celery seed is frequently used worldwide as an appetizer, a vegetable in salads and a spice or flavoring agent (Yang et al., 2019)*.* Also, the seeds and other parts have been used as herbal medication. Various parts of celery, especially its seed, have been used in Ayurvedic medicine. Its useful effects have been shown in the treatment of visceral spasms, gut disease, reduction of flatulence, urinary calculi, and various painful states (Craig, 1999). Celery seed is composed of 2-3% essential oil that can be applied to the flavoring of foods as well as the perfume industry. The oil is rich in both limonene (60%) and selinene (10%*)* (Kitajima et al., 2003). Several chemical compounds are responsible for the celery seed oil aroma, the most biologically active of which are: sedanolide, 3-n-butyl phthalide, and sedanenolide (3-n-butyl-4-5-dihydrophthalide) (Sowbhagya, 2014). Other components of celery seed are listed in Table 1. 

**Table 1:** Chemical compositions of *Apium graveolens *L (celery) seed

**Compounds**
**Fatty acids:** petroselenic, oleic, linoleic, Linolenic, palmitic **Monoterpenes:** D-Limonene, Myrcene, α-pinene, β-pinene, Terpineol, α-Terpinolene, Linalool **Sesquiterpene:** α-selinene, β-Selinene, β-caryophyllene **Phthalides:** sedanenolide, 3-n-butyl phthalide, Sedanolide, sedanonic anhydride **Flavonoids:** apiin, apigenin, Luteolin, Kaempferol **Phenolic acids:** Caffeic acid, Ferulic acid, p-Coumaric acid

Several pharmacological studies illustrated the antioxidant (Wei and Shibamoto, 2007), antibacterial (Powanda et al., 2015), Antiulcerogenic (Baananou et al., 2013), hypolipidemic (Iyer and Patil, 2011) and anti-inflammatory (Powanda et al., 2015) properties of celery. Previous studies demonstrated antihypertensive effects of celery. Administration of various celery seed extracts (hexanic, methanolic, and aqueous-ethanolic) in hypertensive rats induced by deoxycorticosterone acetate, showed antihypertensive effects (Moghadam et al., 2013). The consumption of cooked celery by 2195 Americans caused blood pressure reduction (Chan et al., 2014). Some cardiovascular effects of celery seed can be attributed to phthalide constituents. 3‑n‑butylphthalide (NBP) inhibited oxidative/nitrosative stress in endothelial cells (Li et al., 2009). NBP administration in hypertensive rats inhibited the progression of hypertensive nephropathy (Zhu et al., 2015). In spontaneously hypertensive rat (SHR), administration of NBP showed a hypotensive effect and NBP caused endothelium independent vasorelaxation in the aortic rings of the SHR pre-contracted with phenylephrine (Tsi and Tan, 1997). Recently, the vasodilatory effect of *A. graveolens* in isolated aorta was reported and demonstrated. The effect of celery was endothelium-independent and was probably mediated by calcium antagonism (Jorge et al., 2013), but the roles of extra and intra-cellular calcium in vasodilatory effect of celery seed, were not investigated. In another study, Tashakori-Sabzevar et al. reported that vasorelaxant effect of celery seed extract was endothelium dependent at lower concentrations (Tashakori-Sabzevar et al., 2016), but the roles of calcium and potassium channels in vasodilatory effect of celery seed, were not evaluated. Thus, this study designed to elucidate the roles of endothelium, extra and intra-cellular sources of calcium, and potassium channels in vasodilatory effect of celery seed extract in rats’ isolated aortic rings. 

## Materials and Methods

**Chemical and drugs**

All chemicals used in this study were of analytical grade. Acetylcholine (ACh) (CID:187), phenylephrine hydrochloride (PE) (CID:5284443), NG-nitro-L-arginine methyl ester (L-NAME) (PubChem CID:39836), indomethacin (CID:3715), ruthenium red (RR) (PubChem CID:16218584), heparin (PubChem CID:92044406), tetraethylammonium chloride (TEA) (PubChem CID:6285), glibenclamide (PubChem CID:3488), 4-Aminopyridine (4-AP) (PubChem CID:1727) and diltiazem (PubChem CID:62920) were obtained from Sigma-Aldrich (Germany).

**Plant material and preparation of the extract**

The celery seed was purchased from Pakan Bazr Company (Esfahan, Iran), and identified in the herbarium of Ferdowsi University of Mashhad (voucher No. 152-2016-4). The seeds were ground into a fine powder and rinsed with 2 L hydroalcoholic solution (50% ethanol and 50% water) at room temperature for 48 hr. The extraction solution was filtered, and the solvent was removed by evaporation under vacuum. The extract was kept at 4°C and protected moisture and light. In Krebs solution, the dried extract was dissolved to achieve 0.05, 0.1, 0.25, 0.5, 1, and 2 mg/ml concentrations in organ baths.

**Experimental animals**

The experiment was conducted on male Wistar rats (200-250 g). The rats were kept at 22±1°C temperature with 12 h dark/light cycle and were given tap water for drinking and a standard diet. The experiments were conducted according to the Animal Experimentation Ethics Committee of Mashhad University of Medical Sciences (approval No. 921745).

**Preparation of rat aortas**

The vascular tension was evaluated using organ bath technique as mentioned previously (Niazmand et al., 2014). In brief, the animals were decapitated by guillotine after being anesthetized using ketamine (50 mg/kg, i.p.). The descending thoracic aorta was dissected out quickly and soaked in cold Krebs solution made of the following components (in mM): CaCl_2_ 2.5, MgSO_4_ 1.18, NaCl 118.5, KCl 4.74, NaHCO_3_ 24.9, and glucose 10, and bubbled with a carbogenic mix (5% CO_2_, and 95% O_2_, pH 7.4). The perivascular tissue was removed from the aorta, and broken up into 5-mm ring segments. Any damage to the endothelium was avoided. In 10 ml organ bath having Krebs solution gassed with carbogen, the aortic segments were mounted between two stainless steel ‘L’ shaped hooks at 37ºC. The segments of the vessel were stabilized for 1 hr after a resting tension of 2 g, with altering bath fluid every 15 min to hinder metabolite interference. Isometric transducers linked into a data acquisition system (AD instrument, Australia) were used to record alterations in tension. When required, endothelium was removed by gently rubbing the intimal space using a thin metal rod. To confirm the lack of functional endothelium, PE (10^−6^ M) was used to pre-contract aortic rings; afterward, ACh (10^−5^ M) was added, and contraction was measured; the continued contraction indicated of the lack of functional endothelium.

**Experimental procedure**

**The effect of celery seed extract on aortic contraction induced by KCl** **and PE **

KCl (6×10^−2^ M) and PE (10^−6^ M) were applied to activate a stable contraction in aortic rings with endothelium intact or denuded to assess the vasorelaxant effect of celery seed extract. Celery seed extract was cumulatively added (0.05, 0.1, 0.25, 0.5, 1, and 2 mg/ml), and the relaxation induced by the extract was calculated as a percentage in relation to the contractile response of KCl and PE.

**The effects of indomethacin and L-NAME on celery seed extract induced vasorelaxantion**

To test the involvement of endothelium-dependent mechanisms in the vasorelaxant response, the intact aortic rings were subjected for 30 min to indomethacin (10 µM), a cyclooxygenase inhibitor, or L-NAME (10 µM), a nitric oxide synthase inhibitor, prior to the application of PE (10^−6^ M) to stimulate a stable contraction. Then, the impacts of cumulative concentrations of celery seed extract, were evaluated.

**The** **effect of** **celery seed extract on influx of Ca** ^2+^ **and** **Ca**^2+^** channels**

In the first set of experiments, to investigate the involvement of Ca^2+^ influx in the vasodilator response of celery seed extract, the endothelium-denuded aortic rings were rinsed five times using Ca^2+^-free Krebs solution with EGTA (5×10^−5^ M). This procedure was completed before KCl (6×10^−2^ M) or PE (10^−6^ M) was used, to generate a stable contraction. Ca^2+^ was cumulatively (10^−5^ to 10^-2^ M) added to achieve a concentration–response curve in the presence of 2 mg/ml celery seed extract. To evaluate the roles of voltage-dependent calcium channels in extract-induced relaxation, in the second set of experiments, endothelium-denuded aortic rings were subjected, for 30 min, to diltiazem (10^−5^ M), an L-type Ca^2+^ channel inhibitor, prior to using PE (10^−6^ M) or KCl (6×10^−2^ M) to produce a stable contraction. Vascular relaxation was then conducted by adding 2 mg/ml of celery seed extract.

**The** **effect of** **celery seed extract on intracellular sources of Ca** ^2+^

To assess whether the vasorelaxation of celery seed was associated with inhibiting intracellular Ca^2+^ release, in a set of experiments, endothelium-denuded aortic rings were subjected to ruthenium red (RR) (10^−5^ M), a ryanodine receptor inhibitor (Maggi et al., 1990) and diltiazem (10^−5^ M) 30 min prior to applying PE (10^−6^ M). Subsequently, the celery seed extract (2 mg/ml) was added to induce a relaxation. In a separate set of experiments, endothelium-denuded aortic rings were subjected to heparin (HP) (50 mg/l), an IP3 receptor inhibitor (Mohanty and Li, 2002) and diltiazem (10^−5 ^ M) 30 min prior to applying PE (10^−6^ M) and then, the celery seed extract (2 mg/ml) was added to induce a relaxation.

**The** **effect of** **celery seed extract on K** ^+^ **channels**

To determine the impact of K^+^ channels in the relaxation induced by the extract, in three different sets of experiments, the endothelium-intact aortic rings were subjected to 4-aminopyridine (4-AP) (1 mM), a selective voltage-dependent K^+^ channel blocker, tetraethylammonium chloride (TEA) (5 mM), a nonselective K^+^ channel blocker, or glibenclamide (10^-5^ M), an inhibitor of the ATP-dependent K^+^ channels, for 30 min prior to applying PE (10^−6^ M) to stimulate a stable contraction. Finally, the influences of cumulative concentrations of celery seed extract were evaluated.

**Statistical analysis**

The data is reported as mean±SEM. The EC50, the concentration producing 50% of the maximal response, was determined using nonlinear regression analysis (sigmoidal dose–response with variable slope) by GraphPad Prism (Version 4.0). Statistical analysis was performed with Student’s *t-test* and one-way ANOVA followed by *post hoc* Tukey’s test. Values of p<0.05 were assumed as statistically meaningful.

## Results

**The effect of celery seed on KCl- and PE-contracted aorta**

**Figure 1:**
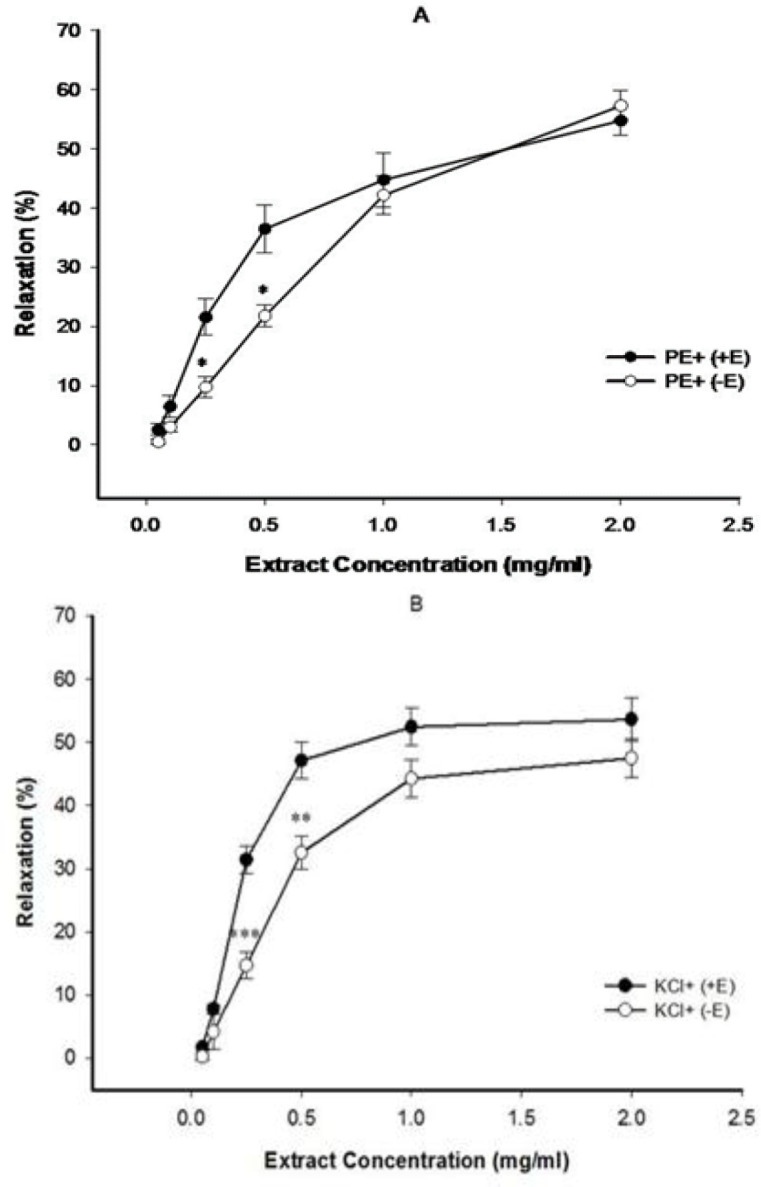
The effect of cumulative concentrations of celery seed extract (0.05, 0.1, 0.25, 0.5, 1 and 2 mg/ml) on PE (10^−6^ M) (A) and KCl (6×10^−2^ M) (B) pre-contracted rat aortic rings with (+E) or without (−E) endothelium. Data are expressed as mean±S.E.M. (n=8). *p<0.05, **p<0.01, and ***p<0.001, compared to (+E) (n=8)

The celery seed extract evoked concentration-dependent relaxation in intact aortic rings pre-contracted using both PE (Figure 1A) and KCl (Figure 1B) with a maximum relaxation of 54.8±2.5% (EC_50_=0.45±0.04 mg/ml) and 53.66±3.38% (EC_50_=0.45±0.036 mg/ml), respectively, at a concentration of 2 mg/ml. The vasorelaxant effects of celery seed at concentrations of 0.025 and 0.5 mg/ml were considerably less than intact aortic rings in denuded aortic rings pre-contracted using PE and KCl.

**The effect of indomethacin and L-NAME on celery seed-induced vasorelaxation**

**Figure 2:**
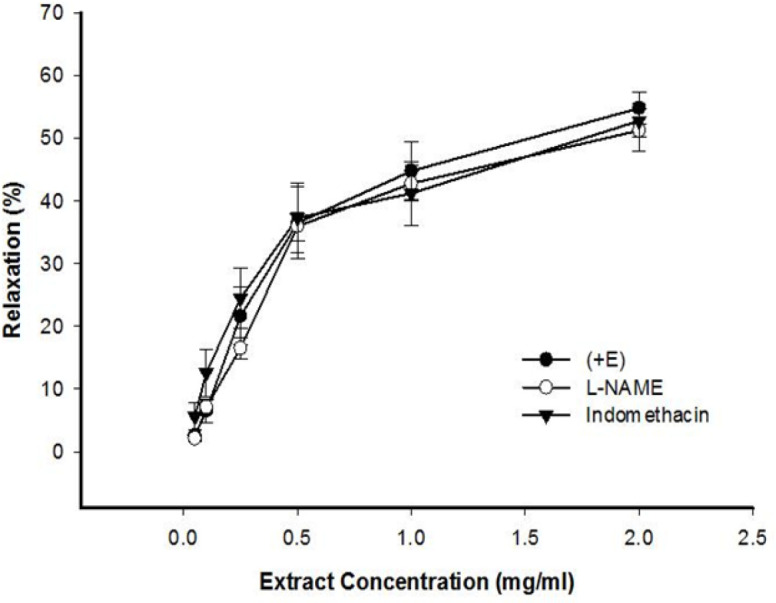
The effect of cumulative concentrations of celery seed extract (0.05, 0.1, 0.25, 0.5, 1 and 2 mg/ml) on PE pre-contracted rat aortic rings with endothelium (+E) and after pretreatment with L-NAME (10 μM) or indomethacin (10 μM). Data are expressed as mean±SEM (n=8)

Figure 2 shows that endothelium-intact aortic rings pretreated with L-NAME and indomethacin, did not indicate the vasorelaxation induced by celery seed.

**The effect of extracellular Ca** ^2+^ **on celery seed -induced vasorelaxation**

**Figure 3:**
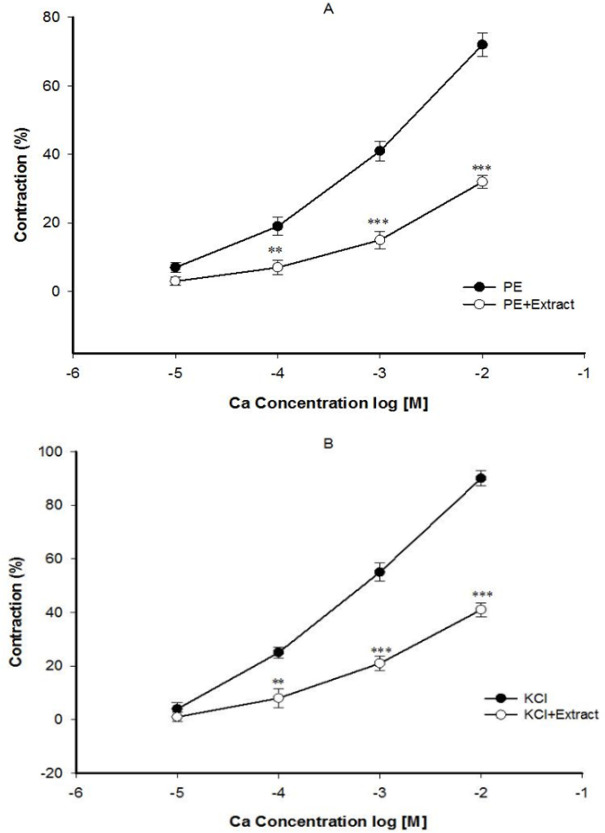
The effect of celery seed extract at 2 mg/ml on the Ca^2+^-induced (0.01-10 mM) contraction of rat aortic rings without endothelium pretreated with PE (10^−6^ M) (A) or KCl (6×10^−2^ M) (B). Data are expressed as mean±SEM (n=8). **p<0.01, and ***p<0.001 compared to control

**Figure 4:**
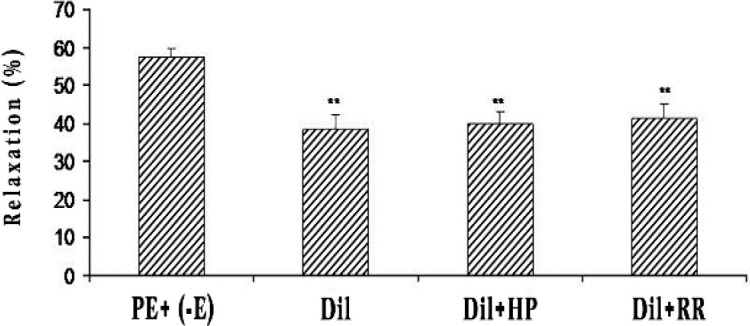
The effect of celery seed extract (2 mg/ml) on rat aortic rings without endothelium contracted with PE (10^−6^ M) in the presence of diltiazem (10^−5^ M) (PE+Dil), after heparin (50 mg/l) (PE+Dil+HP) or ruthenium red (10^−5 ^M) (PE+Dil+RR) pretreatment. Data are expressed as mean±SEM (n=8). **p<0.01 compared to PE+ (-E)

 Pre-incubation of the endothelium-denuded aortic rings with 2 mg/ml of celery seed prevented contraction induced by Ca^2+^ in constricted rings of PE (Figure 3A) and KCl (Figure 3B). In the endothelium-denuded aortic rings pretreated with diltiazem for 30 min and then contracted by PE, the relaxant effect of 2 mg/ml concentration of celery seed was significantly reduced (Figure 4).

**The effect of intracellular sources of Ca** ^2+^ **on celery seed-induced vasorelaxation**

The endothelium-denuded aortic rings pre-incubated with heparin or RR for 30 min in the presence of diltiazem and then contracted by PE, demonstrated heparin and RR did not affect the celery seed vasorelaxation in comparison to diltiazem only (Figure 4).

**The effect of K** ^+^ **channels on celery seed-induced vasorelaxation**

**Figure 5:**
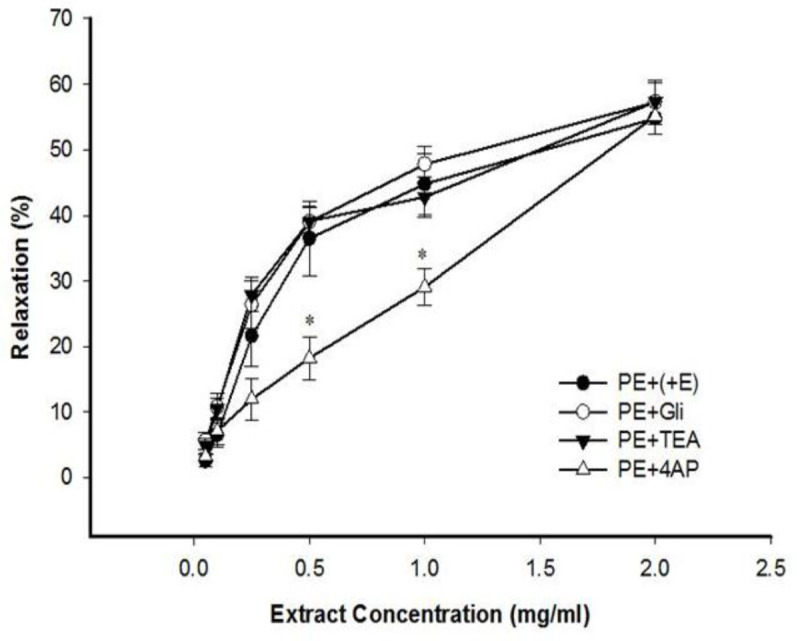
The effect of cumulative concentrations of celery seed extract (0.05, 0.1, 0.25, 0.5, 1 and 2 mg/ml) on rat aortic rings with endothelium (+E) contracted with PE (10^−6^ M), after pretreatment with glibenclamide (10^−5^ M) (PE+Gli), tetraethylammonium chloride (5 mM) (PE+TEA) or 4-aminopyridine (PE+4-AP). Data are expressed as means±SEM (n=8). *p<0.05 compared to PE+(+E)

Endothelium intact aortic rings’ pre-incubation with 4-AP, glibenclamide, or TEA and then contracted by PE, demonstrated that 4-AP significantly attenuated celery seed-induced relaxation in 0.5 and 1 mg/ml concentrations, but TEA and glibenclamide had no effect on celery seed-induced relaxation (Figure 5).

## Discussion

This study illustrated that celery seed concentration-dependently evokes relaxation in PE and KCl pre-contracted rings of the thoracic aorta. Comparing the results of celery seed effect in endothelium-intact and denuded aorta pre-contracted by KCl or PE, indicated the role of endothelium in the preventive impact of the extract (Figure 1A and B). Pretreatment of aortic rings with indomethacin or L-NAME, did not prevent vasorelaxant effect of celery seed extract (Figure 2). These results indicated that additional endothelial pathway(s) may be involved in the vasorelaxant effect of celery seed. Endothelium-derived hyperpolarizing factor (EDHF) could act as the molecule responsible for such vasodilatory effect. At higher concentrations, the endothelium-independent mechanisms of vasodilatory effect of the extract may be prominent. 

The extracellular influx of Ca^2+^ and release of Ca^2+^ from intracellular stores are central components of VSM excitation-contraction coupling. The influx of extracellular calcium through voltage-dependent Ca^2+^ channels (VDCCs) and receptor-operated Ca^2+^ channels (ROCCs) and release of Ca^2+^ from the sarcoplasmic reticulum by activation of inositol triphosphate (IP3) and ryanodine receptors (RYR) (Martinsen et al., 2014) lead to increased intracellular Ca^2+^ and VSMCs contraction. The contraction evoked by KCl mainly results from the cell membrane depolarization and subsequent opening of the VDCCs and influx of extracellular Ca^2+^ (Ratz et al., 2005). Alpha-adrenoreceptors agonist such as PE, evoked aortic contraction by influx of extracellular calcium through ROCCs and release of intracellular Ca^2+^ from the sarcoplasmic reticulum (Niazmand et al., 2014). The latter pathway involves activation of phospholipase C by PE to produce IP3 and diacylglycerol (DAG) from phosphatidylinositol 4, 5-bisphosphate (PIP2). DAG activates the myosin light chain by activating protein kinase C (PKC), and IP3 stimulates releases of Ca^2+^ from the sarcoplasmic reticulum by opening IP3 receptors (Thorneloe and Nelson, 2005).

The impact of celery seed on Ca^2+^ ﬂow within vascular smooth muscle cells was evaluated. The results revealed that celery seed significantly decreased contraction induced by extracellular Ca^2+^ in rings pre-challenged with PE (Figure 3A) and high-KCl (Figure 3B). The vasorelaxant effect of celery seed on PE-induced contraction suggests that celery seed may inhibit ryanodine receptor and/or IP3 pathway and reduce intracellular Ca^2+^, reduce myosin light chain kinase activity through DAG-PKC dependent pathway, and decrease intracellular Ca^2+^ through blocking ROCCs and relaxation of aorta. The finding that celery seed decreases the aortic contraction induced by gradual Ca^2+^ added in a Ca^2+^-free solution in the presence of PE, indicated that ROCCs inhibition by celery seed may be a major mechanism in relaxing the aorta. 

The vasorelaxant effect of celery seed extract in KCl precontracted aortic rings suggests that celery seed inhibits voltage-dependent Ca^2+^ channels (VDCCs). Diltiazem (a blocker of L-type calcium channel), significantly decreased the vasorelaxant impact of celery seed on PE-induced contractions (Figure 4), which indicates the role of VDCCs in vasorelaxant effect of celery seed. Endothelium-denuded aortic rings’ pretreatment with heparin (an inhibitor of IP_3 _receptor) or ruthenium red (a ryanodine receptors inhibitor) with diltiazem, following contraction by PE, did not have a role in the vasorelaxant action of celery seed extract (Figure 4). Thus, the vasodilatory effect of the extract was not mediated by interfering with intracellular Ca^2+^ stores. 

Potassium (K^+^) channels have an important role in VSMCs excitability (Ko et al., 2008) . Efflux of K^+^ ions from VSMCs results in membrane hyperpolarization, causing a decrease in entry of Ca^2+^, and ultimately vasorelaxation (Nelson and Quayle, 1995)*.* Several types of K^+^ channels are expressed in VSMCs such as ATP-sensitive K^+ ^channels (K_ATP_), nonselective K^+^ channels, and voltage-dependent K^+ ^channels (K_v_) (Côrtes et al., 2001).

In this study, the role of potassium channels in celery seed-induced vasorelaxation was evaluated for the first time. The results showed that 4-AP (a K_v_ inhibitor) diminished the relaxation effect of celery seed and reduced the relaxation potency (EC_50_=0.94±0.031 mg/ml), indicating voltage-dependent potassium channel (K_v_) involvement in the vasorelaxant effect of celery seed (Figure 5).

A previous study showed the endothelium-independent vasorelaxant effect of celery attributed to calcium channel (VDCCs and ROCCs) blockade (Jorge et al., 2013). These findings are in line with the present findings, but the vasorelaxant effect of celery seed extract was partly endothelium dependent at lower concentration. Tashakori-Sabzevar et al. reported the vasorelaxant effect of celery seed extract was endothelium-dependent at lower concentrations and NO has a role in vasodilatory effect of celery seed extract (Tashakori-Sabzevar et al., 2016). These results are in accordance of our results, but the reduction of vasorelaxant effect of celery seed extract in the presence of L-NAME was not significant. 

Celery seed is rich in many bioactive compounds which have cardiovascular effects. Butylphthalide is a unique chemical in celery seed (Sowbhagya, 2014). Tsi and Tan investigated the vasorelaxant and hypotensive effects of butylphthalide. They also demonstrated that in aortic rings with intact and denuded endothelium pre-contracted with PE and KCl, butylphthalide could induce relaxation. However, following inhibition of nitric oxide synthase by using L-NAME, the vasorelaxant activity of butylphthalide did not reduce. Butylphthalide also inhibited the cumulative concentration-response curves of phenylephrine and Ca^+2 ^(in CaCl_2 _-free, high KCl medium) (Tsi and Tan, 1997). However, in the current work, extract-induced vasorelaxation was endothelium-dependent at lower concentrations. Accordingly, some part of the vasorelaxant effect of celery seed extract may be attributed to butylphthalide. 

Flavonoids are a large group of polyphenolic substances found in plants which are known for their interesting activity in vascular diseases (Perez-Vizcaino and Duarte, 2010)*. *Apigenin, a celery seed flavonoid, showed an endothelium-dependent vasorelaxant effect in aortic rings that was mediated by influx and release of Ca^2+^, cGMP and nitric oxide (Zhang et al., 2000). Luteolin, another celery seed flavonoid, also showed endothelium-dependent vasorelaxation in aorta (Si et al., 2014). Jiang et al. showed that luteolin endothelium-independently induces relaxation in the thoracic aorta in rats. The involved mechanisms inhibited sarcolemmal Ca^2+^ channels, released intracellular Ca^2+^ stores, and activated K^+^ channels (Jiang et al., 2005). Another celery seed flavonoid, kaempferol, also showed a potent vasorelaxant effect (Duarte et al., 1993). 

The cardiovascular effects of monoterpenes were reviewed (Santos et al., 2011). Linalool (a monoterpene alcohol) showed hypotensive and vasorelaxant effects in rats (Anjos et al., 2013). Menezes et al. showed hypotensive effect of α- pinene, β-pinene and linalool in non-anaesthetized normotensive rats (Menezes et al., 2010). Terpineol (a monoterpene found in the essential oils) also exhibited hypotensive effect and endothelium- dependent vasorelaxation in rat mesenteric artery rings (Ribeiro et al., 2010). Cardoso et al. reported the vasorelaxant effect of limonene in rat superior mesenteric artery (Cardoso et al., 2012). These findings supported our results, the presence of above-mentioned compounds in celery seed extract may also explain the vasorelaxant effect of this plant.

This study demonstrated that celery seed has endothelium- dependent vasorelaxant impacts in aortic rings of rats. The possible involved mechanisms of vasorelaxation include: inhibiting receptor-operated and voltage-dependent calcium channels (ROCCs and VDCCs, respectively), releasing endothelial derived hyperpolarizing factor (EDHF), and activating voltage-dependent potassium channels (K_v_). The pharmacological findings described in the present work may be an important step in the validation of the benefit of celery seed as a phytomedicine in the treatment of hypertension.
